# Relation between switching time distribution and damping constant in magnetic nanostructure

**DOI:** 10.1038/s41598-018-31299-4

**Published:** 2018-09-05

**Authors:** Jung-Hwan Moon, Tae Young Lee, Chun-Yeol You

**Affiliations:** 1 Department of Materials Science and Engineering, Korea University, Seoul, 02841 Korea; 2 New Memory Device and Process Integration Group, SK Hynix, Korea; 3 Department of Emerging Materials Science, DGIST, Daegu, 42988 Korea; 4 Global Center for Bio-Convergence Spin System, DGIST, Daegu, 42988 Korea; 5 Advanced Technology Development Team, Semiconductor R&D Center, Samsung Electronics Co., Ltd., Gyeonggi-Do, 445-701 Korea

## Abstract

It is widely known that the switching time is determined by the thermal stability parameters and external perturbations such as magnetic field and/or spin polarized current in magnetic nano-structures. Since the thermal stability parameter and switching time are crucial values in the design of spin-transfer torque magnetic random access memory, the measurement of the switching time is important in the study of the switching behavior of ferromagnetic nano-structures. In this study, we focus on the distribution of the switching time. Within the limit of a large energy barrier, a simple analytical expression between damping constant and anisotropy field with switching time distribution is obtained and confirmed by numerically solving the Fokker-Planck equation. We show that the damping constant and anisotropy field can be extracted by measuring the full width half maximum of the switching time distribution in the magnetic nano-structure devices. Furthermore, the present method can be applied to not only single nano-structure, but also inhomogeneous nano-structure arrays.

## Introduction

Statistical distributions of measurable physical quantities always have significant physical meanings. Extrinsically, statistical distribution causes measurement error and/or limitations of the accuracy of measurement instruments, in addition to inhomogeneity of samples. Without these extrinsic source ubiquitous finite distributions of the measurable physical quantities can be characterized by the line width, statistical deviation, or full width half maximum (FWHM), and they reveal information about important intrinsic physical quantities such as the atomic layer spacing or the grain size^1^. For example, the peak position (*x*_0_) and the FWHM (Δ*x*) of the X-ray diffraction peak in a *θ* − 2*θ* measurement can give us the atomic layer spacing and information on the grain size^1^. And, the peak position and width of a low angle X-ray reflection gives us some information on the layer thickness and roughness. In magnetic measurements, the peak position and FWHM of ferromagnetic resonance (FMR) spectra are related with the magnetic energy (including anisotropy, exchange, and demagnetization energies) and the Gilbert damping constant^2^, More generally, in any kind of resonance experiment such as one using an RLC circuit or a damped harmonic oscillator, not only the peak position, but also the peak width always has important physical meanings.

Despite the importance of these distributions, most studies on the switching time in magnetic nano-structures, which is essential in modern nano-scale spintronic devices, have focused only on the switching time itself, experimentally^3–13^ and/or theoretically^14–21^. The thermally activated switching time of magnetic nano-structures is determined by the Néel-Arrhenius equation^22^ through the thermal stability parameter and the attempt frequency^23^. Since certain values of the thermal stability parameter ensure digital data storage firmness and write error rate (WER), these values must be carefully examined. Therefore, with/without an external perturbation such as an external magnetic field and/or spin-polarized current, the switching behaviors have been actively studied for decades. However, no studies so far have paid attention to the physical meaning of the switching time distribution.

The damping constant is an important physical property of magnetic materials; it reflects the energy dissipation paths of the given magnetic system. Especially, the critical current for switching in spin-transfer torque magnetic random access memory (STT-MRAM) is proportional to the damping constant^24^; therefore, the characterization of the damping constant of ultra-thin magnetic materials is a challenging issue in the development of STT-MRAM. The damping constant is influenced not only by the intrinsic material properties^25^, but also by many extrinsic sources^26–29^. Especially, ultra-thin magnetic multilayers in magnetic tunneling junctions (MTJ), and spin pumping^30,31^, from adjacent layers, can alter the damping constant significantly. Furthermore, edge effects of the nano-structure can be another source of discrepancy from the bulk values of the damping constant of nano-sized MTJ cells^32^. Therefore, determination of the exact damping constant of each nano-sized MTJ cell will be important in the study of STT-MRAM.

In this study, we find a useful analytic expression for the FWHM of switching time distribution and the damping constant. It is revealed that (i) the FWHM is inversely proportional to the external field, (ii) the damping constant can be extracted from the FWHM, and (iii) the effective anisotropy field can be obtained from the intercept of the relation between reduced external field and FWHM. We analytically solved the Fokker-Planck equation (FPE)^14,32^ to obtain the analytic expression and numerically confirmed the validity and limitations of the presented technique. We also found that our technique might be useful in the determination of the damping constant and the effective anisotropy energy, or of the thermal stability parameters of a nano-sized patterned ferromagnet. Furthermore, it can be applicable not only to single cells, but also to many cell arrays with finite inhomogeneous material parameters, the use of which is much easier and practical in experiments.

## Results

### Switching time for a nano-structure, solution of Fokker Planck equation

The magnetization switching dynamics for nano-structures is well understood and can be obtained by solving the Landau-Lifshitz-Gilbert (LLG) equation. Our concern is the probability of magnetization switching of a free layer of the STT-MRAM; however, solving the stochastic LLG equation requires large computational resources. In this case, it is very convenient to use the Fokker-Planck equation (FPE) because it can statistically describe the switching of the magnetization^14,33^. The FPE is a partial differential equation that describes the flow of the probability density function under certain circumstances. With the thermal fluctuation field, it was reported decades ago that the probability of magnetization switching can be obtained by solving the FPE^33^. Recently, Butler *et al*.^14^ extended the FPE using the spin transfer torque and magnetic field for more general cases. According to Butler’s work^14^, the FPE of the probability distribution of a polar angle as a function of time in the case of uniaxial anisotropy with the limit of single domain approximation can be expressed by

1 $$\frac{\partial \rho (\theta ,\,\tau )}{\partial \tau }=-\,\frac{1}{\sin \,\theta }\frac{\partial }{\partial \theta }({(\sin \theta )}^{2}(i-h-\,\cos \,\theta )\rho (\theta ,\,\tau )-\frac{\sin \,\theta }{2{\rm{\Delta }}}\frac{\partial \rho (\theta ,\,\tau )}{\partial \theta }),$$

where *ρ*(*θ*, *τ*) is the probability that the magnetization is pointing in direction *θ* relative to the film normal (+*z*-axis) at time $\tau =\frac{\alpha \gamma {H}_{k}}{1+{\alpha }^{2}}t$, the dimensionless time scale; Δ = *K*_*u*,*eff*_*V*/*k*_*B*_*T* is the thermal stability factor, where *K*_*u*,*eff*_ is the effective uniaxial anisotropy, *V* is the volume of the sample, *k*_*B*_ is the Boltzmann constant; and *T* is the absolute temperature. Also, $i=\frac{I}{{I}_{Co}}$ and $h=\frac{{H}_{app}}{{H}_{k}}$ are the reduced current and the magnetic field, where *I* is applied current and *I*_*Co*_ is the critical current with only the Slonczewski term, *H*_*app*_ is an applied field (it is always negative to switch the initial + z magnetization) and *H*_*k*_ = 2*K*_*u*,*eff*_/*μ*_0_*M*_*S*_ is an effective anisotropy field including the uniaxial anisotropy and demagnetization energies.

The cumulative switching probability function *P*_*S*_(*τ*) is given by ref.^14^

2 $${P}_{S}(\tau )=\exp (-\frac{{\pi }^{2}}{4W(\tau )}),$$

3 $$W(\tau )=\frac{\exp (2\tau v)}{{\rm{\Delta }}}+\frac{\exp (2\tau v)-1}{v{\rm{\Delta }}},$$

Here, *v* = *i* − *h* − 1, and *v* > 0 implies larger current (negative field) than the switching current (field); switching occurs very rapidly. *v* < 0 means the applied current (field) is smaller than the critical value, so the switching probability is extremely low and only thermally activated switching is possible with long switching time. Here, it must be mentioned that the sign of *h* is always negative to switch the initial +z magnetization. *W*(*τ*) is defined by the ansatz $\rho (\theta ,\tau )=A(\tau )\exp (-{\theta }^{2}/W(\tau ))$, where *A*(*τ*) and *W*(*τ*) are related with the normalization condition of probability^14^. Therefore, the physical meaning of *W*(*τ*) is the width of the distribution of *θ* at the given time. When *τν* ≫ 1, and there is strong current (field) or long time, *W* is very large; then *P*_*S*_ → 1, which implies that almost 100% switching occurs. It must be mentioned that Eqs (2) and (3) are approximations, such that numerical calculation of Eq. (1) is required to obtain solutions for general cases.

**Figure 1:**
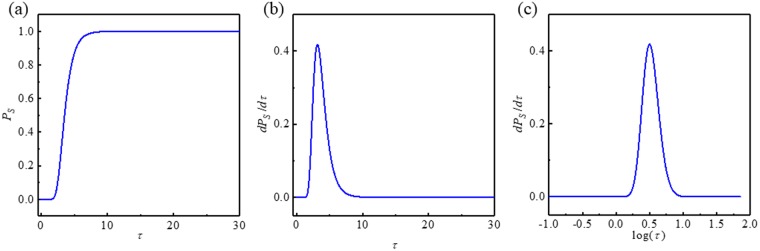
Typical example of full numerical solution of the FPE. (**a**) Cumulative switching probability *P*_*S*_, (**b**) *dP*_*S*_/*dτ* as a function of dimensionless time *τ*. This is a log-normal distribution and (**c**) *dP*_*S*_/*dτ* vs. $\mathrm{log}(\tau )$ shows normal distribution.

Figure 1(a) shows a typical example of a cumulative switching probability *P*_*S*_(*τ*) when *h* = −1.5 and *i* = 0 with *α* = 0.01; Δ = 68.25 at 300 K (corresponding to *H*_*k*_ = 8000 Oe). The corresponding *v* = 0.5 and τ = 1.41 × 10^9^(*Hz*)⋅*t*(*s*). The time derivative $\frac{d{P}_{S}(\tau )}{d\tau }$ is shown in Fig. 1(b); it follows the log-normal distribution. We depict it again in log-scale, $\mathrm{log}(\tau )$, to obtain a normal distribution function, as shown in Fig. 1(c).

Let us find the peak position and FWHM of $\frac{d{P}_{S}(\tau )}{d\tau }$. By taking *τ* derivative of Eq. (2),

4 $$\frac{d{P}_{S}}{d\tau }=\frac{{\pi }^{2}{\rm{\Delta }}{v}^{2}(v+1){e}^{2\tau v-\frac{{\pi }^{2}{\rm{\Delta }}v}{4(v+1){e}^{2\tau v}-4}}}{2{((v+1){e}^{2\tau v}-1)}^{2}}.$$

In order to find the peak position, we have to find a value of *τ*_0_ that will satisfy the condition of $\frac{{d}^{2}{P}_{S}({\tau }_{0})}{d{\tau }^{2}}=0$. After tedious algebra, we find

5 $${\tau }_{0}=\frac{\mathrm{log}(\frac{\sqrt{{\pi }^{4}{{\rm{\Delta }}}^{2}{v}^{2}+64}+{\pi }^{2}{\rm{\Delta }}v}{8(v+1)})}{2v}.$$

The FWHM can be determined by its definition

6 $$\frac{{\pi }^{2}{\rm{\Delta }}{v}^{2}(v+1){e}^{2\tau v-\frac{{\pi }^{2}{\rm{\Delta }}v}{4(v+1){e}^{2\tau v}-4}}}{2{((v+1){e}^{2\tau v}-1)}^{2}}=\frac{{e}^{\frac{1}{8}(-\sqrt{{\pi }^{4}{{\rm{\Delta }}}^{2}{v}^{2}+64}+{\pi }^{2}{\rm{\Delta }}v-8)}(\sqrt{{\pi }^{4}{{\rm{\Delta }}}^{2}{v}^{2}+64}+8)}{{\pi }^{2}{\rm{\Delta }}}.$$

Due to the complex form of Eq. (6), FWHM cannot be easily obtained analytically, only a numerical solution is available. Let us assume a large barrier case (Δ > 40), and *τν* ≫ 1. We can make proper approximations of Eq. (4), and obtain

7 $$\frac{d{P}_{S}}{d\tau }\approx \frac{{\pi }^{2}{\rm{\Delta }}{v}^{2}{e}^{-\frac{{\pi }^{2}{\rm{\Delta }}v}{4(v+1){e}^{2\tau v}}-2\tau v}}{2(v+1)}.$$

From Eq. (7), the peak position and height of the peak are

8 $${\tau }_{0}\approx \frac{\mathrm{log}(\frac{{\pi }^{2}{\rm{\Delta }}v}{4(v+1)})}{2v}.$$

9 $${(\frac{d{P}_{S}}{d\tau })}_{\max }\approx \frac{-2v}{e}$$

Equation (8) is not in exactly the same form as the results of Eqs (2.12) and (2.14) in ref.^14^; however, it has a similar dependence on *v*. If we ignore the logarithmic term, we can roughly say that ${{\tau }_{0}}^{-1}$ is proportional to *H*_*app*_ when only the magnetic field is applied. More details will be provided later.

The FWHM (=Δ*τ*) of the peak is given by

10 $${\rm{\Delta }}\tau =\frac{W(-\frac{1}{2e})}{2v}-\frac{{W}_{-1}(-\frac{1}{2e})}{2v}\approx \frac{1.223}{v}.$$

Here, *W*_*k*_ is the *k*-th branch of the Lambert *W* function, and *e* is Euler’s number. Eq. (10) is the central result of our study. It implies that the dimensionless FWHM of the switching time distribution (Δ*τ*) is inversely proportional to *v*, and we can rewrite Eq. (10) as

11 $${\rm{\Delta }}\tau =\frac{\alpha \gamma {H}_{k}}{1+{\alpha }^{2}}{\rm{\Delta }}t\approx \frac{1.223}{v}=\frac{1.223}{i-h-1}=\frac{1.223}{I/{I}_{C0}-{H}_{app}/{H}_{k}-1}.$$

With the assumption of small α ≪ 1 and the magnetic field only case (*I* = 0, *H*_*app*_ < −*H*_*k*_, *h* < −1), Eq. (11) can be simplified for Δ*t* to give the FWHM in units of seconds,

12 $${\rm{\Delta }}t\approx \frac{1}{\alpha \gamma }\frac{-1.223}{{H}_{app}+{H}_{k}}.$$

13 $$\frac{1}{{\rm{\Delta }}t}\approx \frac{-\alpha \gamma }{1.223}({H}_{app}+{H}_{k}).$$

This is the main finding in this study. If we measure Δ*t*^−1^ as a function of *H*_*app*_, we can obtain a linear line: the slope is $\frac{-\alpha \gamma }{1.223}$ and the intercept of the *y*-axis is $\frac{-\alpha \gamma }{1.223}{H}_{k}$. Therefore, we can determine the damping constant of the ferromagnetic nano-structure from the switching time distribution measurement. Furthermore, we can also determine *H*_*k*_ from the intercept.

### Numerical solution of Fokker Planck equation

#### Determination of *α* and *H*_*k*_ from the switching time distribution

**Figure 2:**
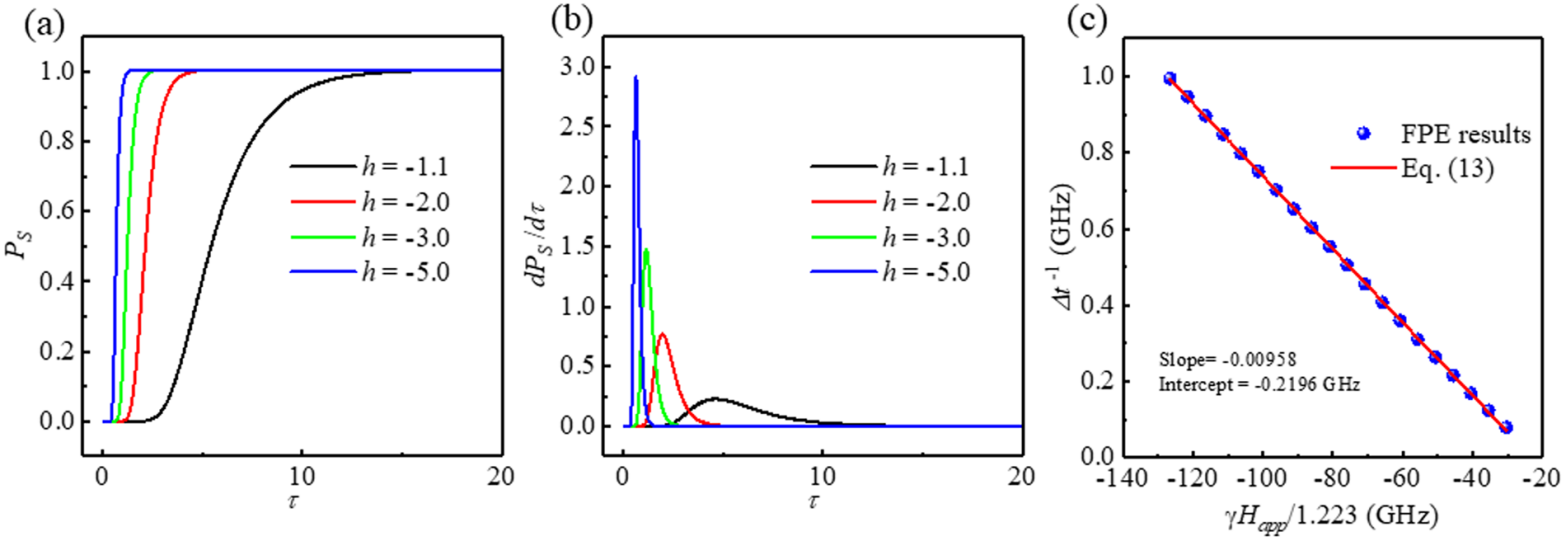
(**a**)*P*_*S*_, (**b**) *dP*_*S*_/*dτ* for various *h* (=−1.1 ~ −5.0) and (**c**) Δ*t*^−1^ as a function of *γH*_*app*_/1.223 with the linear fitting result of Eq. (13).

We were able to depict the numerically calculated *P*_*S*_(*τ*) and *dP*_*s*_(*τ*)/*dτ* by numerically solving the FPE. The material parameters and dimensions of the sample were the following values: Δ = 60, *α* = 0.01^34,35^ and volume of the cell = *π* 30 × 30 × 1 nm^3^, *M*_*s*_ = 1.0 × 10^6^ A/m; corresponding *H*_*k*_ is 1.4 × 10^5^ A/m in Fig. 2(a,b) for various *h* (from −1.1 to −5.0). The stronger the magnetic field, the shorter the peak switching time, as can be clearly seen in Fig. 2(a,b). From the log-normal distribution of *dP*_*s*_/*dτ*, we can numerically obtain Δ*t*^−1^ and plot it as a function of *γH*_*app*_/1.223 to find the slope and intercept, as shown in Fig. 2(c), with linear fitting. The slope of the linear fit is −0.00958 (=−*α*_*fit*_), and *H*_*k*,*fit*_ = 1.264 × 10^5^ A/m is obtained from the intercept of −0.2196 GHz (=−*γH*_*k*,*fit*_/1.223). The error from the input value is acceptable (*α*_*fit*_/*α*_*input*_ = 0.96 ~ 4% error and *H*_*k*,*fit*_/*H*_*k*,*input*_ = 0.90 ~ 10% error). Despite the crude approximation of Eq. (13), the agreement is excellent. From Fig. 2(c), we can confirm that the analytic expression Eq. (13) is well matched with the full numerical results.

Equation (13) is somewhat similar to the well-known relation between the line width and the damping constant in FMR experiments, ${\rm{\Delta }}H={\rm{\Delta }}{H}_{ext}+\frac{4\pi }{\sqrt{3}}\frac{\alpha f}{\gamma }$, where Δ*H*, Δ*H*_*ext*_, and *f* are the peak to peak line width, extrinsic line width from inhomogeneity of the samples, and measurement frequency, respectively^36,37^. Generally, in FMR measurement, *α* is determined from the slope of Δ*H* as a function of *f*. However, the FMR signal is proportional to the volume of the sample; it is extremely difficult to carry out FMR measurement for ultra-thin nano-sized samples.

#### Effect of various Δ, *H*_*k*_, and temperature

**Figure 3:**
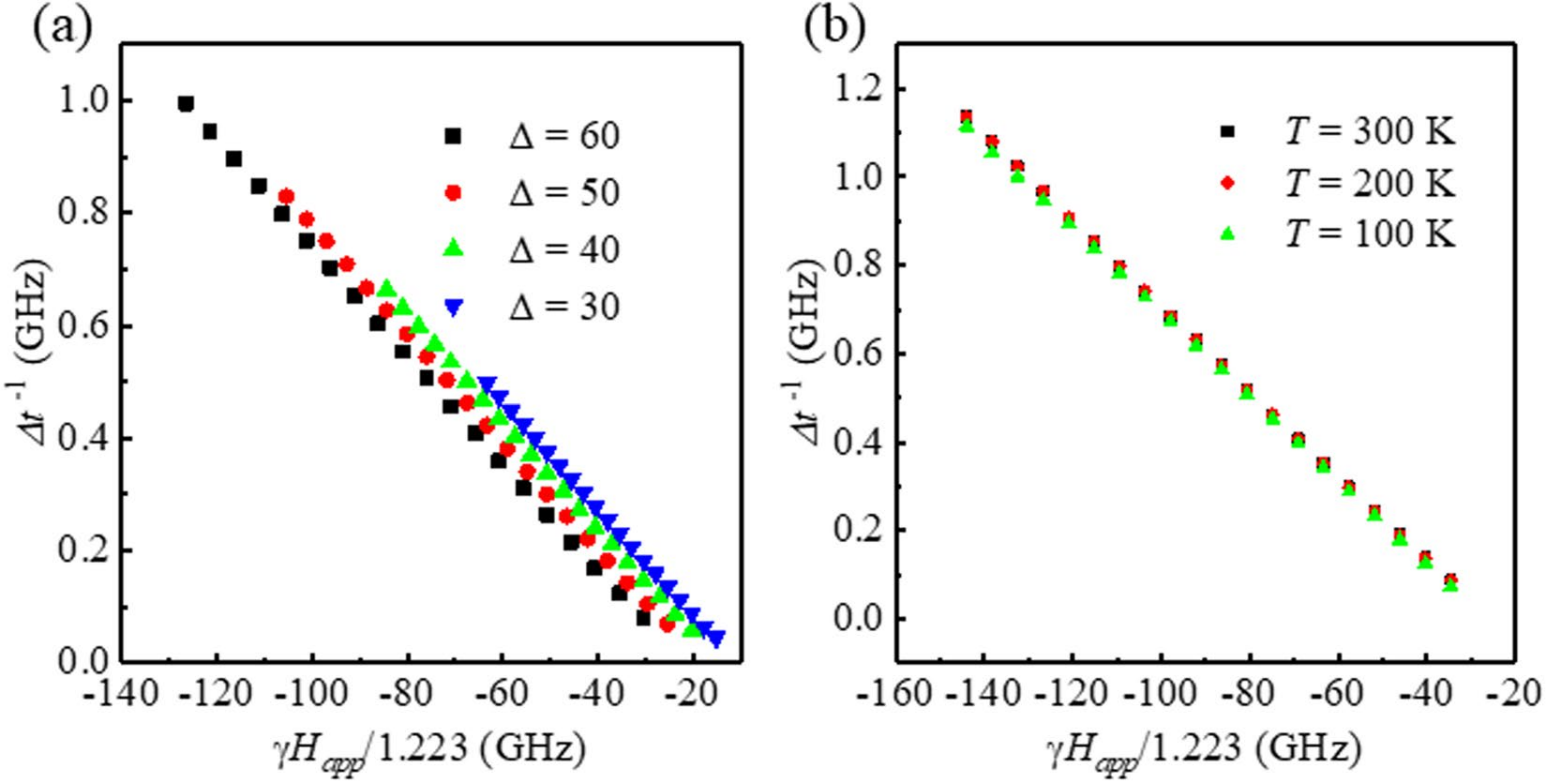
Δ*t*^−1^ as a function of *γH*_*app*_/1.223 for various (**a**) Δ = 30, 40, 50, and 60 with constant T = 300 K, (**b**) T = 100, 200, and 300 K with constant *H*_*k*_.

Figure 3(a) shows the numerically calculated Δ*t*^−1^ as a function of *γH*_*app*_/1.223 for various Δ (=30, 40, 50, 60) at 300 K. With the same *α* input values, *H*_*k*_ varies with Δ$(=\frac{{\mu }_{0}{M}_{s}{H}_{k}V}{2{k}_{B}T})$ for a constant temperature. From Eq. (13), it can be easily understood that the slopes are the same, and only the intercepts change with Δ or *H*_*k*_. The error of *α* is only 4~5%, while the error of *H*_*k*_ is rather larger (10~16%). When Δ is larger, the error is smaller. Practically, the value of Δ required for STT-MRAM application is >60; the typical error in this method is about 5% for *α* and 10% for *H*_*k*_.

We depicted Δ*t*^−1^ again as a function of *γH*_*app*_/1.223 for various temperatures (100, 200, and 300 K) with the same materials parameters, as shown in Fig. 3(b). Since we varied only the temperature with constant *H*_*k*_ (=1.59 × 10^5^ A/m), all Δ*t*^−1^ plots collapse to a single line. This is also well explained by Eq. (13), in which there is no dependence on temperature. However, physically, it sounds very strange. It seems that the switching time distribution is independent of the temperature. When we take a close look at the derivation process, the Δ dependence disappears during the approximation with the assumption of Δ ≫ 1. The truth however is that there is a very small temperature dependence in the numerical solutions; Δ*t*^−1^ is almost insensitive to the temperature. Therefore, Eq. (13) works well with a wide range of temperature and is useful to determine the temperature dependent value *α* if the assumption of Δ ≫ 1 is valid. Experimentally, Eq. (13) requires only measurements of the switching time distribution as a function of the applied field; we do not need any other material parameters for the determination of *α*; this will be a very strong point of the presented technique.

#### Inhomogeneous nano-structure array

So far, we have considered only switching time distributions for a single nano-structure. The switching time distribution can be obtained by repeated switching measurement for a single cell. However, generally, nano-size single cell measurement is not experimentally easy. Practically, switching measurement of many cells is experimentally easier because of the larger signal; furthermore, many-cell measurements give us a statistical switching time distribution. However, when we consider many cells simultaneously, the inhomogeneity of each cell must be addressed. For many possible reasons, nano-size cell anisotropy and size will vary from cell to cell. Such inhomogeneity of individual cells will influence the measurement of Δ*t*^−1^. The sample to sample variation of *H*_*k*_ or Δ is a serious problem in practical STT-MRAM production. The variation of each cell, *δH*_*k*_ or *δ*Δ, is the main source of switching voltage (current) distribution; it must be monitored and controlled to reduce write bit error in STT-MRAM.

**Figure 4:**
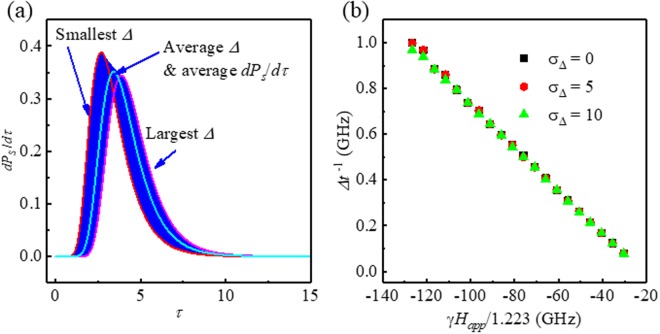
(**a**) Blue colored area is overlap of 100 *dP*_*S*_/*dτ* plots for different Δ values with *σ*Δ = 10; the two red plots represent minimum and maximum Δ. The sky blue solid line is average *dP*_*S*_/*dτ* for all Δ s. (**b**) Δ*t*^−1^ values as a function of *γH*_*app*_/1.223 are shown for *σ*Δ = 0, 5, and 10.

**Figure 5:**
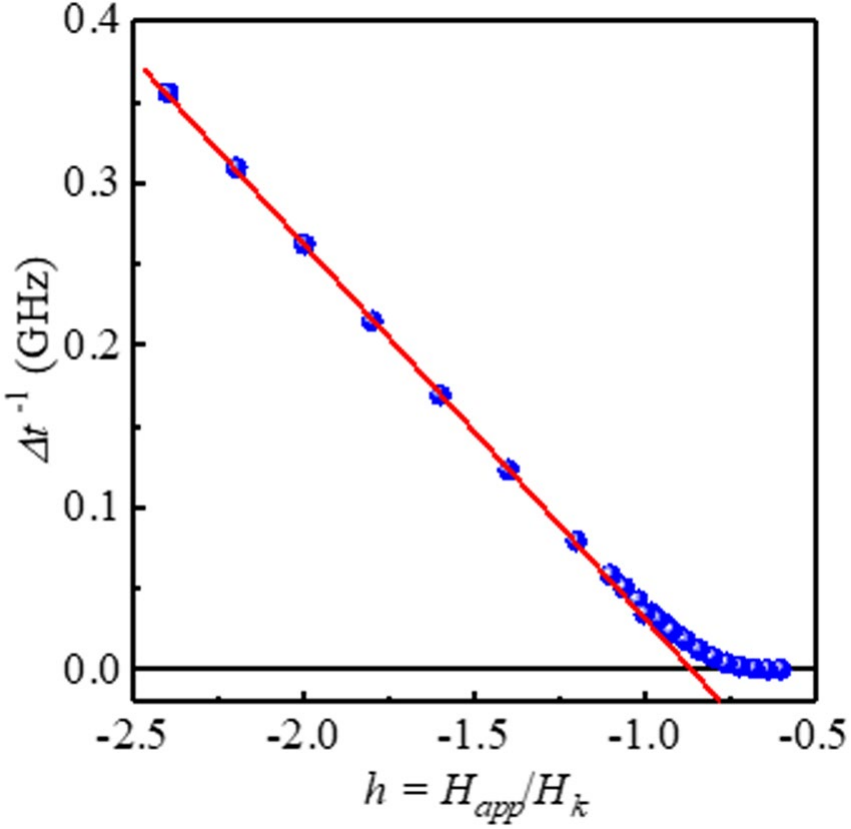
Δ*t*^−1^ as a function of *h* = *H*_*app*_/*H*_*k*_. The deviation from the linear dependence increases when |*h*| < 1. The symbols represent the full numerical solution of the FPE, and the red solid lines are the linear fitting results of Eq. (13).

We assumed a finite normal distribution of Δ values with a standard deviation of *σ*Δ. Each *dP*_*s*_/*dτ* is plotted in Fig. 4(a) for *σ*Δ = 10 and Δ = 60. The peak and FWHM of *dP*_*s*_/*dτ* are found to change with various Δ. After calculating *dP*_*s*_/*dτ* for each Δ, we obtained a weighted averaged *dP*_*s*_/*dτ* with an assumption of normal distribution. Since we assumed a normal distribution, the averaged *dP*_*s*_/*dτ* is identical to ${(d{P}_{s}/d\tau )}_{{\sigma }_{{\rm{\Delta }}}=60}$. Therefore, surprisingly enough, the ratios of Δ*t*^−1^ to *H*_*appl*_ are almost identical for cases of *σ*Δ = 0, 5, and 10, as shown in Fig. 5(b), and the obtained values of *α* and *H*_*k*_ are the same as those of the single cell case. Here, *σ*Δ = 0 is the single Δ value or single cell case. This result seems at a glance counterintuitive; however, it is physically correct. The presented measurement technique is solid for the inhomogeneity of Δ of each cell; this implies that the variations of *H*_*k*_ and volume of each cell do not affect the determination of the value of *α* in the presented technique. Since Eq. (13) is linear to *α* and *H*_*k*_, we can always obtain the average values of *α* and *H*_*k*_ for the cell array.

#### Validity and limitations of Eq. (13)

**Figure 6:**
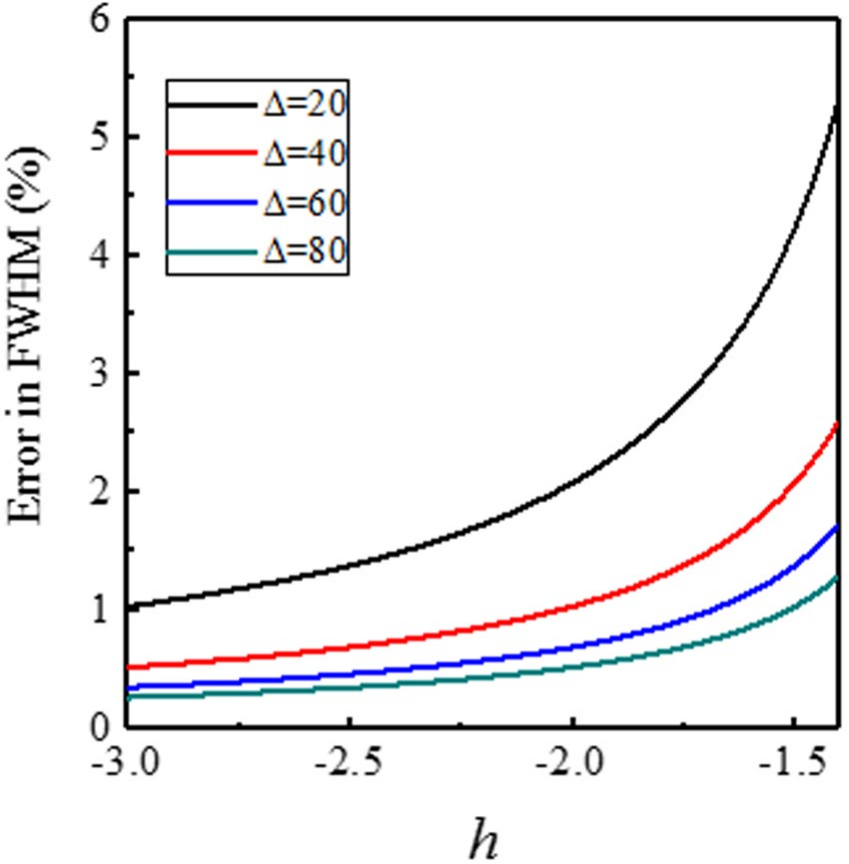
Difference between Eqs (6) and (10) assuming a larger energy barrier. The difference between the exact and approximated solutions decreases with larger thermal stability factor by increasing applied field.

We derived Eq. (13) with the assumption of *τν* ≫ 1. We were interested in a large enough case of Δ > 50, because the thermal stability must be guaranteed for STT-MRAM applications. Furthermore, the *ν* ≫ 1 condition implies a large enough STT current and/or magnetic field to ensure STT and/or field driven switching processes. We have to emphasize that Eq. (13) is not valid for thermally activated switching, where *v* ≤ 0. Figure 5 shows the limitations of the presented technique. For large |*h*|, the linearity of Δ*t*^−1^ is excellent, while in the small |*h*| region, Δ*t*^−1^ deviates from linear behavior. In our numerical calculations (for Δ = 60), |*h*| > 1.25 is required, and a larger |*h*| gives better fitting results. For experiments, the linearity of the curve of Δ*t*^−1^ vs. *h* will be a criterion of the validity of the measurement. Figure 6 shows the difference between the exact solution of Eq. (6) and the approximated solution of Eq. (10), assuming a larger energy barrier. The difference between the exact and approximated solutions is smaller for larger thermal stability factors. The difference between Eqs (6) and (10) is sufficiently small (<1%) for large Δ and large *h*.

## Discussion

We theoretically and numerically study the relation between damping constant and anisotropy field with the FWHM of the switching time distribution for the magnetic nano-structure. The physical meaning of the switching time distribution has so far not been considered in most studies, and so we reveal that the switching time distribution contains important physical information such as the line width of the FMR measurement. We derive approximate analytic expressions of the full-width half-maximum (FWHM, Δ*t*) of the switching time distribution for the case of a high thermal barrier. We find that the measured Δ*t*^−1^, as a function of the external field, shows linear dependence; the damping constant *α* and the effective anisotropy field can be extracted using the damping constant *α* and the intercept. The FWHM of the switching time distribution is in a range of nanoseconds; this is expected to be large enough to be resolved by state-of-the-art experimental techniques. Our prediction allows us to estimate the damping constant and the effective anisotropy field simultaneously from the switching time distribution of a nano-size patterned sample.
